# Copenhagen Magnetic Personalized Accelerated Brain Circuit Therapy (CoMPACT) trial: study protocol for a randomized, double-blinded, sham-controlled, three-armed, parallel-group trial of transcranial magnetic stimulation for treatment-resistant depression

**DOI:** 10.3389/fpsyt.2026.1937455

**Published:** 2026-10-02

**Authors:** Armita Faghani Jadidi, Iman Ibrahim, Lasse Christiansen, Mikkel Malling Beck, Vytautas Labanauskas, Björn Sigurdsson, Marlene Gomes Antunes, Thomas Thomsen, Simon Hjerrild, Axel Thielscher, Maj Vinberg, Hartwig Roman Siebner, Poul Videbech

**Affiliations:** 1 Danish Research Centre for Magnetic Resonance, Department of Radiology and Nuclear Medicine, Copenhagen University Hospital - Amager and Hvidovre, Copenhagen, Denmark; 2 Center for Neuropsychiatric Depression Research, Mental Health Center Glostrup, Copenhagen University Hospital - Amager and Hvidovre, Copenhagen, Denmark; 3 Department of Neuroscience, Faculty of Medical and Health Sciences, University of Copenhagen, Copenhagen, Denmark; 4 Department of Health Technology, Technical University of Denmark, Kgs Lyngby, Denmark; 5 Department of Clinical Medicine, Faculty of Medical and Health Sciences, University of Copenhagen, Copenhagen, Denmark; 6 The Early Multinodular Prevention and Intervention Research Institution (EMPIRI), Mental Health Centre, North Zealand Hospital, Northern Zealand, Denmark; 7 Department of Neurology, Copenhagen University Hospital Bispebjerg and Frederiksberg, Copenhagen, Denmark

**Keywords:** accelerated iTBS (aiTBS), intermittent theta burst stimulation (iTBS), major depressive disorder (MDD), randomized controlled trial (RCT), transcranial magnetic stimulation (TMS), treatment-resistant depression (TRD)

## Abstract

**Background:**

Treatment-resistant depression (TRD), characterized by poor response to conventional antidepressant therapies, remains a major clinical challenge. Repetitive transcranial magnetic stimulation (TMS) of the left dorsolateral prefrontal cortex (dlPFC) has emerged as a promising alternative. Employing intermittent theta burst stimulation (iTBS), TMS protocols have yielded rapid antidepressant effects in recent accelerated treatment studies. These findings warrant further investigation in larger samples to confirm efficacy and in-depth investigation into the underlying mechanisms. Moreover, other cortical sites may also represent suitable targets for accelerated iTBS treatment of TRD.

**Methods:**

This randomized, double-blind, sham-controlled trial will assess the efficacy, feasibility and mechanisms of a novel, personalized, and accelerated iTBS protocol for TRD, targeting two left hemispheric regions, the left dlPFC and inferior parietal lobule (IPL), both characterized by anticorrelated functional connectivity with the subgenual anterior cingulate cortex. 78 adults (aged 18–95 years) will be recruited from Danish mental health centers and randomly assigned to one of three groups: (1) active iTBS of the left dlPFC, (2) active iTBS targeting of the left IPL, or (3) sham iTBS at either site. The intervention includes five daily sessions over five consecutive days. Targeting informed by group-level functional connectivity will be combined with individualized coil placement and stimulation dosing using electric field modeling based on whole-brain anatomical magnetic resonance imaging (MRI). Antidepressant efficacy will be evaluated using the 6-item Hamilton Depression Rating Scale as the primary outcome, assessed at an early post-intervention follow-up (F1) within three days after the final stimulation session. Multimodal post-treatment assessments, including MRI, will be conducted within five days after the intervention (T1). A four-week follow-up (T2) will assess the durability of clinical and cognitive outcomes. Neurophysiological mechanisms will be investigated using electroencephalography as well as structural and functional MRI.

**Discussion:**

By testing a novel stimulation protocol and evaluating the efficacy of a new parietal target, this study will help advance the development of innovative, circuit-based interventions for TRD, with potential implications for broader applications across neuropsychiatry.

**Clinical trial registration:**

https://clinicaltrials.gov/, identifier NCT06895863.

## Introduction

### Background and rationale

Major depressive disorder (MDD) affects approximately 15-20% of all individuals during their lifetime (1). It deeply impacts the quality of life and work performance and elevates risks of unemployment, somatic comorbidities, and suicide (2). Up to one-third of patients with MDD develop treatment-resistant depression (TRD) as they fail to respond to or cannot tolerate currently available antidepressant interventions such as psychotherapy, pharmacotherapy, or electroconvulsive therapy (2–4). MDD is recognized as a disorder of “brain circuits” (5) and standard treatments often fall short of reinstating normal circuit function. This underscores the need for novel therapeutic approaches that specifically address the circuit-level disruptions characteristic of TRD.

Transcranial magnetic stimulation (TMS) is a non-invasive method for modulating neural activity in the human brain (6). Depending on the stimulus intensity, a TMS pulse can elicit synchronized excitation or inhibition of cortical neurons and connected brain regions. When given repetitively as continuous trains or bursts, TMS may induce lasting neuroplastic effects that extend beyond the stimulation session (7, 8). Repetitive TMS (rTMS) targeting the left dorsolateral prefrontal cortex (dlPFC) is an established treatment for MDD. High frequency (>5 Hz) rTMS for TRD was approved by the U.S. Food and Drug Administration (FDA) in 2008. Since then, many studies have proved the efficacy of this treatment for TRD (9). Patterned rTMS stimulation of left dlPFC, especially intermittent theta burst stimulation (iTBS), has subsequently been shown to be non-inferior to conventional 5Hz rTMS for the treatment of MDD (10) and received FDA approval in 2018 (11), offering comparable efficacy in a substantially shorter session time. Although the precise mechanism of action remains unclear, converging evidence suggests that antidepressant effects may be mediated through a modification of functional connectivity between the left dlPFC and the subgenual anterior cingulate cortex (sgACC) (12–14).

This functional connectivity framework informed the development of the Stanford Accelerated Intelligent Neuromodulation Treatment (SAINT) protocol for TRD by Cole and colleagues (15, 16). Using pre-treatment resting-state functional MRI, the investigators identified, for each patient, the left dlPFC site exhibiting the strongest anticorrelation with the sgACC, enabling individualized targeting for an accelerated iTBS protocol. Participants received active or sham 50-Hz iTBS delivered as ten sessions per day (18,000 pulses/day) over five consecutive days. The active group demonstrated a 53% mean reduction in depressive severity, compared with an 11% reduction in the sham group, with treatment effects sustained at four-week follow-up. While highly promising, these findings require replication in larger cohorts, and the original protocol demanding daily schedule may limit broader clinical implementation. Moreover, the neurobiological mechanisms underlying its antidepressant efficacy remain insufficiently understood. Moreover, other cortical sites may also represent suitable targets for accelerated iTBS treatment of TRD. Motivated by these considerations, the present trial investigates a more practical personalized accelerated iTBS protocol and examines its proposed neurophysiological mechanisms.

## Objectives

The CoMPACT stimulation protocol used in the present investigation introduces several important modifications over recently proposed accelerated iTBS protocols. These include:

1. A novel accelerated iTBS protocol
  - Five iTBS sessions per day with bursts at 130 Hz: Stimulation using 50–200 Hz bursts has been shown to induce long-term potentiation (LTP)-like plasticity in the motor cortex (17, 18). It has been suggested that high-frequency burst TBS gives rise to a maximal postsynaptic calcium influx in stimulated cortical neurons, a key cellular trigger of LTP. We hypothesize that high frequency burst iTBS (HF-iTBS) will induce strong LTP-like plasticity in the cortical target, and that five daily sessions will produce a sufficient “acceleration” effect in terms of therapeutic efficacy. CoMPACT trades off “acceleration” for “tolerability,” giving patients the opportunity to rest in the afternoon while still delivering a high stimulation dose. The reduced number of iTBS sessions per day relative to the SAINT protocol (15, 16) also increases clinical feasibility and scalability in hospital settings.
  - Group-level Connectivity-informed targeting and personalized stimulation dosing: The stimulation dose will be personalized using electric field (E-field) modeling informed by individual structural brain MRI data. Target regions will be identified based on group-level probabilistic functional connectivity data from another cohort (as described in objective 2) and transformed into each participant’s native space. The individual target will be located at varying cortical depths due to interindividual variation. Stimulation intensity will be adjusted for cortical depth and individual anatomy based on E-field modeling, using a state-of-the-art in-house developed dosing approach based on SimNIBS (19, 20). The goal is to deliver stimulation intensity such that E-field in the targeted region corresponds to the E-field induced in the precentral motor (M1) hand knob by TMS of the primary hand area. The coil position and angle will be optimized using E-field modeling to induce the maximum E-field in the intended region while minimizing co-stimulation of other adjacent brain areas.
2. Testing clinical efficacy of CoMPACT for a new cortical target
  Building on the coordinate identification approach described earlier, we selected stimulation targets based on large-scale probabilistic functional connectivity data from the Human Connectome Project (21) (N ≈ 1000, https://db.humanconnectome.org/) For the dlPFC, stimulation targets will be defined as the coordinates showing the greatest functional anticorrelation with the sgACC, which is consistent with prior TMS studies (22). For the IPL, which represents a novel target in this context, the site will be selected as the coordinate that shows the strongest positive functional connectivity with the dlPFC target, while simultaneously residing within a region that is strongly anticorrelated with the sgACC. This connectivity-based approach ensures that IPL stimulation functionally engages a network consistent with the antidepressant mechanism typically associated with dlPFC targeting. Furthermore, patients with multiple sclerosis and comorbid depression often present with cortical lesions in the IPL, further supporting the IPL as a potential target for antidepressant intervention (23).
  The primary clinical hypothesis is that active CoMPACT stimulation targeting the left dlPFC will be more effective in reducing depressive symptoms than sham stimulation. The secondary efficacy hypothesis is that active CoMPACT stimulation targeting the left IPL will be more effective in reducing depressive symptoms than sham stimulation.
3. Exploring TMS mechanisms by multimodal functional brain mapping Online electroencephalography (EEG) recording will be carried out shortly before, during, and after several single iTBS sessions to explore how the novel iTBS protocol with within-burst repetition rates of 130 Hz functionally engages the stimulated and connected cortex areas. Based on a recent TMS study (24), we hypothesize that a single CoMPACT session will mitigate prefrontal or parietal oscillatory theta activity, depending on the stimulation site. In addition to resting EEG recordings, TMS-evoked potentials (TEPs) will be also recorded before and after the intervention to examine how stimulation modulates cortical inhibition and excitation in target regions (25), and whether baseline cortical reactivity predicts clinical outcome.
  Furthermore, resting-state and reward-based task fMRI before and after the 25 iTBS sessions will enable us to test the hypothesis that CoMPACT exerts its therapeutic effect by normalizing functional activation in key structures of the reward network and improving resting-state connectivity between the sgACC and the orbitofrontal cortex (26, 27).

## Methods: patient and public involvement, trial design

### Trial design

This is a randomized, double-blind, sham-controlled, parallel-group trial designed to assess the effectiveness of two active CoMPACT stimulation protocols targeting either 1) the left dlPFC or 2) the left IPL for the treatment of TRD, compared to 3) a sham CoMPACT protocol. Participants will be allocated in equal proportions to these three groups. Within the sham group, stimulation will be further divided equally between sham targeting the left dlPFC (50%) and the left IPL (50%).

The study follows a superiority framework. The primary efficacy comparison tests whether active CoMPACT stimulation targeting the dlPFC results in a greater reduction in depressive symptoms than sham stimulation. The comparison between active IPL stimulation and sham stimulation constitutes a secondary efficacy comparison.

### Patient and public involvement

Patients or members of the public were not directly involved in the design of the trial. However, the structure of the intervention schedule, outcome measures, and follow-up visits were informed by prior clinical experience with patients receiving TMS for depression in psychiatric services.

During the early implementation phase of the trial, participants’ feedback regarding the practical aspects of the intervention schedule and study procedures will be collected informally to assess feasibility and participant burden. Such feedback may inform minor practical adjustments to study procedures if needed, particularly during the initial recruitment phase. Any substantial protocol modifications will follow the established amendment procedures and be communicated to the relevant ethics committees and regulatory authorities.

## Methods: participants, interventions and outcomes

### Study setting

The CoMPACT trial is a multicenter study conducted in Denmark. Participants will be screened clinically and recruited from the Mental Health Services of Copenhagen. Treatment sessions and multimodal functional brain mapping procedures are coordinated and performed at the Danish Research Centre for Magnetic Resonance *(DRCMR).*

### Eligibility criteria

Eligible participants will be adult inpatients or outpatients aged 18–95 years with a diagnosis of moderate to severe major depressive disorder (MDD). Diagnosis of a single or recurrent depressive episode will be established according to ICD-10 criteria (28), and confirmed using the Mini International Neuropsychiatric Interview (MINI) (29).

Participants must have a score >25 on the self-reported Major Depression Inventory (MDI) (30), and meet criteria for treatment-resistant depression, defined as insufficient response to at least two antidepressant medications from different pharmacological classes (e.g., selective serotonin reuptake inhibitors, serotonin–norepinephrine reuptake inhibitors, tricyclic antidepressants, or monoamine oxidase inhibitors) administered at adequate dose and duration during the current episode, as judged by the investigator. The duration of the current depressive episode must be between two months and four years. The four-year upper limit was selected based on prior studies indicating that longer duration of the current depressive episode is associated with poorer TMS treatment outcomes (31, 32). This criterion should be regarded as a pragmatic methodological strategy to improve sample homogeneity rather than as a clinical assertion that TMS is ineffective in patients with longer episodes.

Exclusion criteria include a history of neurological disorders affecting the brain (e.g., dementia, epilepsy, stroke, or other progressive neurological conditions) and a diagnosis of schizophrenia or other psychotic disorders (except psychotic depression). Individuals with psychotic symptoms or acute suicidal risk that may compromise safe participation or transport to study visits will be excluded. Compulsory psychiatric admission or treatment within the past three months will also constitute grounds for exclusion.

Additional exclusion criteria include receipt of electroconvulsive therapy (ECT) or non-response to TBS during the current depressive episode, or current harmful substance use or dependence according to ICD-10 criteria that may interfere with outcome assessment (verified by urine testing). Any medical or psychiatric condition deemed by the investigator to compromise participant safety or protocol adherence will result in exclusion.

Female participants of childbearing potential must not be pregnant (confirmed by urine testing), breastfeeding, or unwilling to use effective contraception throughout the study period. Individuals with contraindications to MRI or TMS (33–35), or those who decline disclosure of clinically relevant MRI findings, will be excluded.

### Who will take informed consent?

Potentially eligible participants will be contacted by a project-responsible medical doctor to arrange the screening visit. The purpose of the visit will be to inform the candidate about the project and to assess the eligibility. The visit will be arranged for admitted patients in the department where they are treated. For outpatients, the visit can occur in the patient’s outpatient clinic or the research center. The visit will take place in a quiet, undisturbed room, and sufficient time is offered for questions. If deemed potentially eligible based on preliminary information, they will receive verbal and written information about the study from a study-affiliated medical doctor. Following this, written informed consent will be obtained prior to any study-related procedures. Participants will be given up to 24 hours to consider participation.

Following consent, the study physician will access the patient’s electronic medical records, in accordance with Danish legislation. The screening will include a review of current and previous psychiatric and medical diagnoses, treatment history (including medication, TMS or ECT), and relevant information regarding contraindications for MRI or TMS (e.g., history of seizures, implants). This access will also allow for documentation of participation and reporting of any clinically significant findings during the trial.

### Additional consent provisions for collection and use of participant data and biological specimens

No additional consent provisions are required because no biological specimens will be collected, and no ancillary use of participant data is planned.

## Interventions

### Explanation for the choice of comparators

Consented participants will be randomized to receive one of three interventions: active CoMPACT targeting the left dlPFC, active CoMPACT targeting the left IPL, or sham stimulation to either region. The dlPFC is included as a standard target with well-established efficacy in TMS treatment for depression, serving as a reference condition. The IPL and sham conditions are included to assess the clinical potential of a novel stimulation target and to control placebo effects, respectively, allowing for evaluation of both treatment efficacy and target specificity.

### Intervention description

The CoMPACT intervention consists of five daily sessions over five consecutive weekdays, totaling 25 HF-iTBS or sham sessions. Each HF-iTBS session delivers 1,800 pulses over approximately 10 minutes, with a 50-minute inter-session break. HF-iTBS comprises bursts of three pulses at 130 Hz, delivered every 200 ms, at 100% of the active motor threshold from the First Dorsal Interosseous (FDI) muscle. As detailed above, the stimulation intensity is adjusted for each participant based on the depth difference between the stimulation target (IPL or dlPFC) and M1. Stimulation will be delivered using a B65-Cool-A/P coil connected to a MagPro XP Orange Edition stimulator (MagVenture, Farum, Denmark). The B65-Cool-A/P coil is a butterfly-shaped coil with active cooling that can be used for both active and placebo stimulation. The MagPro XP Orange Edition stimulator can deliver high-frequency biphasic stimulus trains (2–5 pulses) up to 1000 Hz with minimal intensity drop-off. The TMS coil position will be continuously monitored with a Localite neuronavigation system based on individual T1-weighted MRI scans (Localite, Bonn, Germany). The stimulation targets, the left dlPFC and left IPL, are defined in MNI space at x,y,z-coordinates (-42, 38, 31) and (–55, –41, 49), respectively, based on our prior analyses from the HCP cohort as described in the objectives section.

Participants in the sham group will receive the same iTBS protocol as the active groups. However, the stimulation coil will be flipped, with the placebo side facing the scalp. This orientation, achieved by rotating the coil along its longitudinal axis, prevents the delivery of a biologically effective magnetic field while preserving the auditory and tactile experience of stimulation.

All stimulation sessions will be administered by trained physicians or research staff trained in transcranial magnetic stimulation safety procedures. Operators undergo protocol-specific training prior to trial initiation to ensure consistent stimulation delivery and adherence to safety guidelines.

### Criteria for discontinuing or modifying allocated interventions

The allocated intervention will be discontinued if continued stimulation is considered clinically unsafe, including in the event of a TMS-induced seizure, emergence of acute suicidal risk requiring clinical intervention, serious stimulation-related adverse event, or a newly emerging contraindication to TMS. Intervention may also be discontinued at the request of the participants. Any adverse effects or safety concerns will be evaluated by the study team, including psychiatric specialists, in accordance with institutional safety procedures. Intervention discontinuation may also occur if participants are unable to complete the baseline structural MRI, thereby preventing target projection and individualized dosing, or if they demonstrate non-compliance with the intervention schedule, including repeated missed sessions despite contact attempts. All participants will retain the right to withdraw from the trial at any time without providing a reason. In cases of stimulation-related discomfort, intensity and coil position may be individually adjusted using real-time E-field modeling (SimNIBS RT) to optimize tolerability while maintaining target engagement.

### Strategies to improve adherence to interventions and to promote participant retention and complete follow-up

Inpatients will remain at the hospital during the day and be accompanied by a study nurse or physician between the treatment site and the inpatient ward, if admitted. For outpatients, transportation to and from the hospital will be arranged by the research team. During breaks between sessions, all participants will have access to a study nurse or physician. To support participant retention and ensure complete follow-up, the research team will maintain regular contact with participants, offering reminders and practical assistance where needed. Participants who cancel with short notice will be contacted by phone and encouraged to continue the intervention.

Participants who discontinue the trial before receiving the intervention will not undergo further trial assessments. For participants who discontinue after initiation of the intervention, follow-up assessments may be completed where feasible and clinically appropriate, unless the participant withdraws consent for further data collection.

### Relevant concomitant care permitted or prohibited during the trial

Participants can continue their usual pharmacological treatment and/or psychotherapy throughout the study period. No specific restrictions will be placed on standard clinical care during trial participation. However, the study physician and investigators will assess all concomitant treatments for compatibility with the protocol. The dose of antidepressant medication must remain stable during the trial participation and should not be changed unless clinically necessary, in which case the change will be documented and reviewed.

### Provisions for post-trial care

All participants are covered by the national healthcare insurance system in Denmark (Patientforsikringen).

After completion of the trial, participants will resume standard clinical care, including medication and psychotherapy as determined by their treating clinicians. Any participant who experiences harm related to study participation will receive medical care in accordance with national regulations and institutional policies.

Participants who do not achieve remission, defined as HAMD-17 score <7, either immediately following the CoMPACT intervention or at the T2 follow-up visit will be referred for standard TMS treatment, which is approved for routine clinical use in psychiatric settings.

## Outcomes

Main assessments will be conducted at baseline (T0), an early post-intervention follow-up (F1) within three days after the final intervention session focused on the Hamilton Depression Rating Scale assessment, a post-treatment visit (T1) within five days after the intervention period including multimodal MRI, cognitive testing, and additional clinical measures, and a four-week follow-up visit (T2) to assess durability of clinical and cognitive outcomes.

### Primary outcome variables

The primary outcome of the trial will be the change in depression severity, assessed using the 6-item Hamilton Depression Rating Scale (HAMD-6) (36), a brief clinician-rated scale with strong sensitivity to change in core depressive symptoms. Depression severity will be measured at baseline (T0), within three days following the final intervention session (F1), and at four-week follow-up (T2).

The primary analysis will evaluate the difference in change in HAMD-6 score from T0 to F1 between the active dlPFC stimulation group and the sham group. The corresponding comparison between the active IPL stimulation group and the sham group will constitute a secondary efficacy analysis. Additional analyses will examine the persistence of clinical effects at T2.

### Secondary outcome variables

Secondary outcomes will evaluate the broader clinical and functional effects of the intervention across post-intervention and four-week follow-up (T2). To provide a clear hierarchy for interpretation, HAMD-17 depression severity, clinical response, and remission will be considered key secondary clinical outcomes and will be assessed at the early post-intervention follow-up (F1) and four-week follow-up (T2). The remaining secondary outcomes will provide supportive information across affective, anxiety, sleep, functional, cognitive, safety, tolerability, expectancy, and blinding domains and will be interpreted accordingly. These outcomes are grouped into four domains:

Affective and anxiety symptoms

- Depression severity and clinical status: HAMD-17, including clinical response (≥50% reduction in HAMD-17 score from baseline) and remission (HAMD-17 <7).
- Clinician-rated mood changes: Young Mania Rating Scale (YMRS (37)) and daily mood ratings using the Visual Analogue Mood Scale (VAMS), assessed before and after each intervention day in addition to T1 and T2 visits.
- Self-reported symptoms: anhedonia with Snaith-Hamilton Pleasure Scale (SHAPS) (38), anxiety using Overall Anxiety Severity and Impairment Scale (OASIS) (39), and well-being by the World Health Organization-Five Well-Being Index (WHO-5) (40)

Sleep and functioning

- Sleep quality: Pittsburgh Sleep Quality Index (PSQI) (41).
- Psychosocial functioning: Functioning Assessment Short Test (FAST) (42).

Cognitive outcomes

- Subjective cognitive complaints: Cognitive Complaints in Bipolar Disorder Rating Assessment (COBRA) (43)
- Objective cognitive performance: Screen for Cognitive Impairment in Psychiatry (SCIP) (44), Stroop Color and Word Test (SCWT) (45), Trail Making Test A & B (46), and Nine-Hole Peg Test (9HPT) (47).

Safety, tolerability, and treatment expectancy

- Side effects: Aarhus Side Effect Assessment Questionnaire (ASAQ) (48).
- TMS-related sensations: TMS Sensation Questionnaire (TMSens-Q) (49).
- Treatment expectancy and blinding: Treatment Expectation Questionnaire (TEX-Q) (50) and treatment allocation guess (TMS-AG).

### Exploratory outcome variables

Exploratory outcomes will characterize neurophysiological and neuroimaging changes associated with the intervention. Participants will undergo multimodal MRI at baseline (T0) and post-intervention (T1) to assess structural, microstructural, and functional connectivity alterations within stimulation-targeted networks. MRI acquisition includes structural, diffusion, and functional MRI sequences. The fMRI protocol incorporates both resting-state and task-based paradigms. The task-fMRI uses a grip-force paradigm developed and implemented within our research group, designed to engage reward, motor, and affective networks: participants perform left- or right-hand grip responses to symbolic cues (Go trials) or withhold responses (No-Go trials), while ignoring task-irrelevant emotional faces. Correct responses are financially rewarded, with changing reward probabilities across the task to modulate reward circuit activity relevant to depression pathophysiology (51, 52).

Neurophysiological outcomes will be obtained using EEG to evaluate acute and cumulative cortical responses to HF-iTBS. EEG is recorded on Days 1 and 5 of the intervention and includes resting-state (rs)-EEG and TEPs immediately before and after stimulation, as well as EEG acquired during stimulation (iTBS-EEG). Analyses will assess changes in oscillatory power (53, 54), connectivity and inhibitory/facilitatory TEP components implicated in depression (e.g., N45, P60, N100) (55). An in-house tool (EStiMo) (56) will be used to quantify immediate cortical reactivity during stimulation. Electrooculogram (EOG) is recorded throughout all EEG recordings to facilitate eye motion artifact detection in the EEG time series data.

Furthermore, heart rate variability (HRV) and three-lead electrocardiography (ECG) will be recorded during stimulation to index autonomic engagement and heart–brain coupling as additional markers of target-specific network modulation (57).

### Participant timeline

The full overview of outcome measures and their assessment timeline is presented in **Figure 1**.

**Figure 1:**
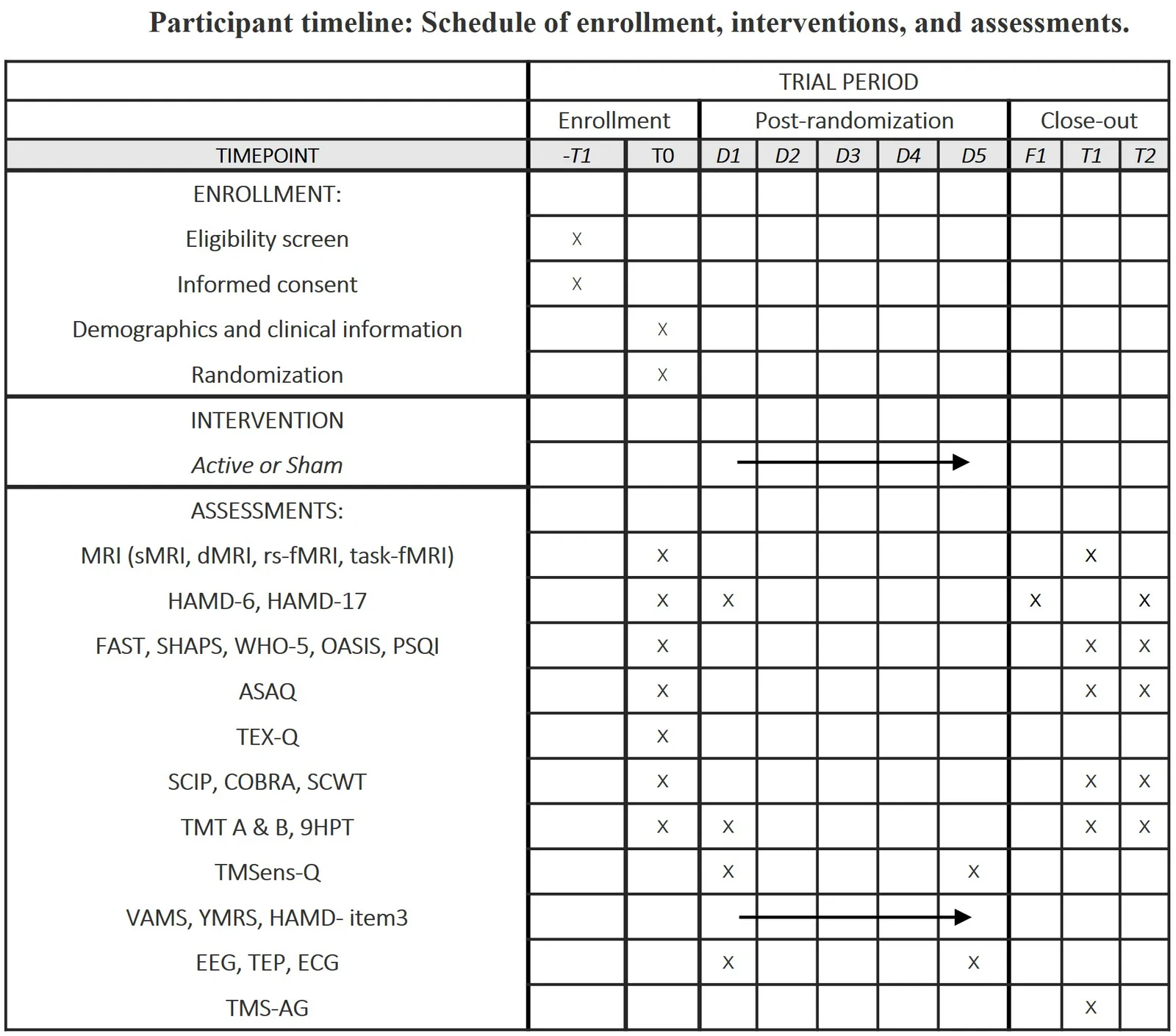
Participant timeline: Schedule of enrollment, interventions, and assessments. Schedule of study procedures across enrolment, baseline, intervention week (D1 -D5), and follow-up visits. "X" indicates that the assessment or procedure is performed at the specified time point. −T1, pre-screening; T0, baseline; D1 -D5, intervention days 1 -5; F1, immediate follow-up 1; T1 -T2, post-intervention follow-ups. 9HPT, Nine-Hole Peg Test; ASAQ, Adverse Symptom Assessment Questionnaire; COBRA, Cognitive Complaints in Bipolar Disorder Rating Assessment; dMRI, diffusion magnetic resonance imaging; ECG, electrocardiography; EEG, electroencephalography; FAST, Functioning Assessment Short Test; HAMD-6/17, Hamilton Depression Rating Scale (6- and 17-item); MRI, magnetic resonance imaging; OASIS, Overall Anxiety Severity and Impairment Scale; PSQI, Pittsburgh Sleep Quality Index; rs-fMRI, resting-state functional magnetic resonance imaging; SCIP, Screen for Cognitive Impairment in Psychiatry; SCWT, Stroop Color -Word Test; SHAPS, Snaith -Hamilton Pleasure Scale; sMRI, structural magnetic resonance imaging; task-fMRI, task-based functional magnetic resonance imaging; TEP, TMS-evoked potentials; TEX-Q, Treatment Experience Questionnaire; TMS-AG, TMS Adverse Events Questionnaire; TMSens-Q, TMS Sensation Questionnaire; TMT A & B, Trail Making Test Parts A and B; VAMS, Visual Analogue Mood Scale; WHO-5, World Health Organization Well-Being Index; YMRS, Young Mania Rating Scale.

The trial will include the following key time points: eligibility screening, a baseline assessment (T0), an intervention period, and three follow-up assessments (F1, T1 and T2). Participants begin with a baseline visit (T0) that includes MRI, clinical, and cognitive assessments. This will be followed by the intervention phase. An immediate follow-up assessment (F1) is conducted within three days after the final intervention session and includes the primary clinical outcome (HAMD-6). A post-treatment visit (T1), including multimodal MRI, cognitive testing, and additional clinical assessments, will be performed within five days after the intervention period. A second follow-up visit (T2) will take place four weeks later and includes clinical and cognitive assessments only. A graphical overview of the full study timeline is presented in **Figure 2**.

**Figure 2:**
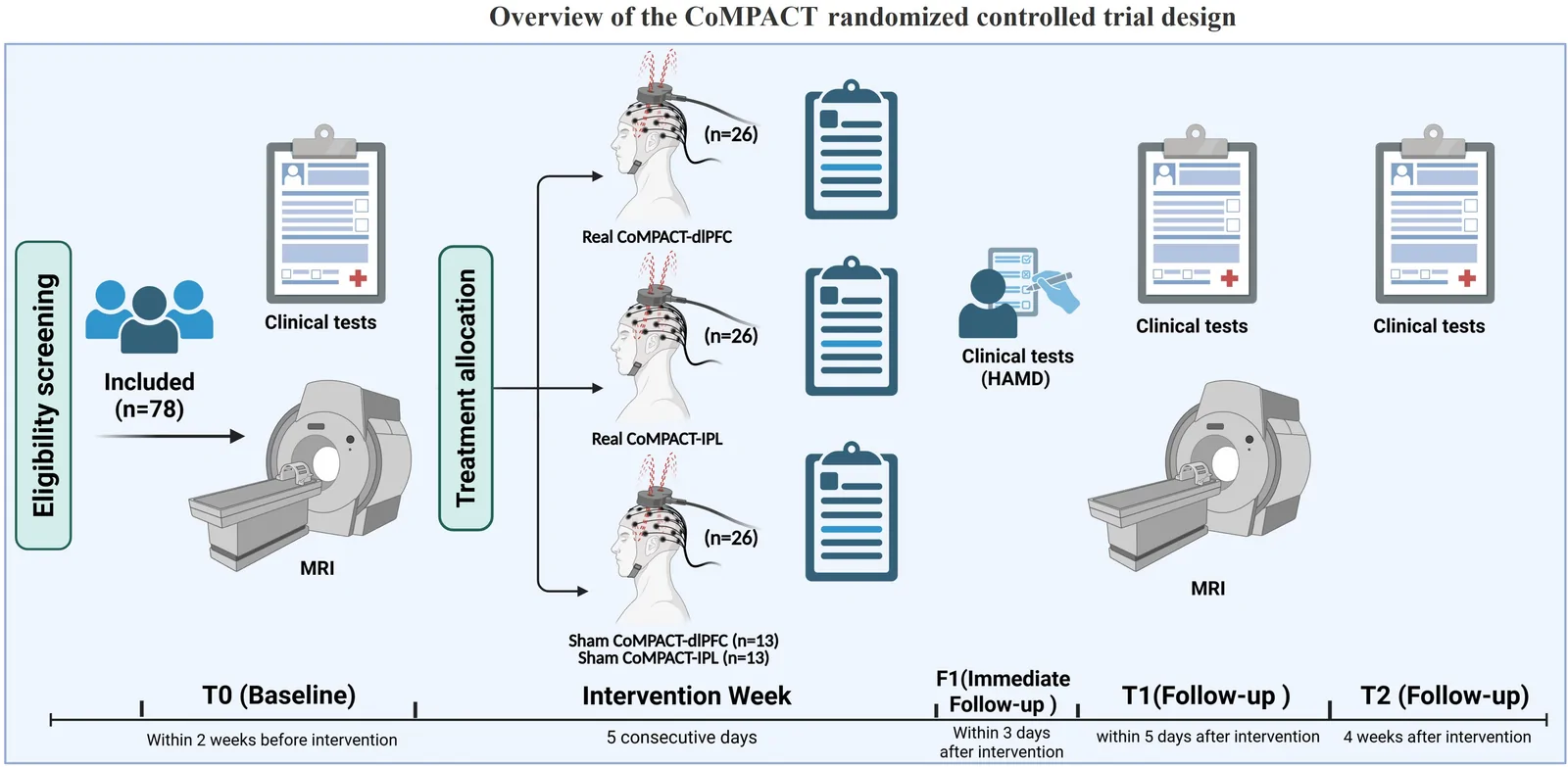
Overview of the CoMPACT randomized controlled trial design. Following eligibility screening and baseline assessments (T0), 78 participants are randomized to one of three groups: real CoMPACT-DLPFC (n=26), real CoMPACT-IPL (n=26), or sham stimulation (n=26; sham DLPFC n=13, sham IPL n=13). The intervention consists of five consecutive treatment days. Clinical assessment and MRI are performed at baseline, immediately after the intervention week, and at follow-up visits within 5 days (T1) and 4 weeks (T2) post-treatment. Abbreviations: CoMPACT, Copenhagen Magnetic Personalized Accelerated Brain Circuit Therapy; dlPFC, dorsolateral prefrontal cortex; HAMD, Hamilton Depression Rating Scale; IPL, inferior parietal lobule; MRI, magnetic resonance imaging. Created in BioRender. Faghani, A. (2026) https://BioRender.com/caqqi28.

### Sample size

The sample-size calculation was based on the primary superiority comparison between active dlPFC stimulation and sham stimulation. Assuming an effect size of Cohen’s d = 0.85, 18 evaluable participants per group are required to achieve 80% power at a one-sided significance level of α = 0.05. The assumed effect size is informed by prior accelerated iTBS studies reporting large treatment effects in TRD populations (15, 16). The primary outcome is change on the HAMD-6 scale, with a minimal clinically important difference set at 3 points (58, 59). To accommodate an anticipated dropout rate of 30%, we plan to enroll 26 participants per group, yielding a total of 78 across the three study arms. The IPL-versus-sham comparison constitutes a secondary efficacy comparison and was not used to determine the sample size. This sample size should also be sufficient to explore neural correlates of the CoMPACT interventions (exploratory outcome) and to assess the feasibility of implementing these protocols in a Danish hospital setting. Sample size calculations were performed using GIGA-calculator online statistical software (https://www.gigacalculator.com/calculators/power-sample-size-calculator.php).

### Recruitment

The participants will be recruited from the Mental Health Centers of the Capital Region of Denmark and through digital recruitment platforms. Healthcare professionals from the participating centers will be informed about the project via clinical conferences, information meetings, posters, recruitment announcements, etc. Additionally, we will use a pop-up window (a tool designed in the Danish medical documentation system (Sundhedsplatformen) informing a healthcare professional that a patient may be eligible for the trial. The health professional can contact research staff to refer a possible participant or dismiss the pop-up window. Research staff will also visit the recruiting centers or contact them by phone regularly to screen for potential participants. Finally, to enhance public visibility and facilitate direct contact with potential participants, digital recruitment platforms such as Trialtree (https://trialtree.com/) will also be used.

## Assignment of interventions: allocation

### Sequence generation and Implementation

A total of 78 participants will be randomized centrally by the coordinating center using an in-house developed Python-generated algorithm written in Python. Randomization was performed in two steps with variable block sizes to minimize predictability: first, participants were allocated in a 1:1:1 ratio to groups A, B, and C (26 per group); second, those in group C were further randomized equally into subgroups C1 and C2 (13 each). Final group labels were recorded to W, X, Y, and Z with a key file to maintain allocation concealment. The randomization sequence was generated using the in-house algorithm by a research assistant who is not directly involved in the trial and was stored on secure, access-restricted servers at the host institution.

### Concealment mechanism

Only the research coordinator will have access to the allocation sequence and key file. Staff responsible for administering the stimulation procedures will be informed of participant allocation one working day prior to the start of the intervention during an in-person meeting for lab preparation. Enrolling personnel and outcome assessors will not have access to the randomization sequence.

## Assignment of interventions: blinding

### Who will be blinded

All participants, clinical assessors and other study staff except treatment providers are and will continue to be blinded. Participants will be instructed not to discuss stimulation sensations with study staff or other patients. All TMS sessions will use similar stimulation coils, without indication of active or sham condition. To match the subjective experience of iTBS between real and sham, CoMPACT uses a dedicated (A/P) stimulation coil with both active and sham functionality generating the same sound level regardless of the stimulation character. The stimulator displays no indication of condition assignment. Additionally, all patients will wear earplugs to dampen the acoustic discharging artifact caused by iTBS. Patients, clinical assessors, and investigators are blinded until the trial has been completed.

### Procedure for unblinding if needed

Should serious adverse events or worsening of depressive symptoms occur after inclusion, the blinding might be lifted to ensure appropriate clinical care. In case of discontinuation of the trial, i.e., due to unexpected adverse events, the principal investigator from the coordinating site can acquire the randomization key from access-restricted servers at the host institution for each subject.

## Data collection and management

### Plans for assessment and collection of outcomes

#### Questionnaires and clinical measures

Questionnaire-based data will be collected and managed using REDCap electronic data capture tools hosted at The Capital Region of Denmark (RegionH) (60, 61). Self-report instruments will be administered digitally via secure REDCap survey links. If digital completion is not feasible, validated paper versions will be provided and subsequently entered into electronic case report forms (eCRFs) in REDCap. Clinician-rated assessments will be performed by trained raters who have completed calibration sessions and inter-rater reliability checks prior to study initiation.

#### MRI data acquisition

Brain MRI data will be acquired at DRCMR on a 3 Tesla Siemens MAGNETOM Prisma scanner using standard operating procedures at baseline (T0) and follow-up (T1). Structural imaging will include a T1-weighted MPRAGE sequence (0.9 mm isotropic voxels, TR = 2700 ms, TE = 3.7 ms, duration ≈ 6 min) and a T2-weighted SPACE sequence (0.9 mm isotropic voxels, TR = 2000 ms, TE = 347 ms, duration ≈ 4 min). Diffusion MRI will be acquired using a multiband EPI protocol (1.8 mm isotropic voxels, TR = 2541 ms, TE = 64 ms, 75 axial slices) with b-values of 0 and 2500 s/mm². A high-angular-resolution dataset with 129 diffusion directions will be collected with anterior–posterior (AP) phase encoding, accompanied by a brief reverse phase-encoding reference scan (posterior–anterior, PA). Total acquisition time is approximately 6 minutes.

Functional MRI will include both a validated grip-force task and resting-state acquisition. BOLD-sensitive images will be collected with 2.5 mm isotropic voxels, 56 interleaved axial slices, multiband acceleration factor 2, and TR = 1880 ms. The task-fMRI run (~10 min) will be performed once, and resting-state fMRI (~8 min) will be performed in two runs with opposite phase-encoding directions (AP/PA) to enable distortion correction.

#### EEG

Neurophysiological outcomes will be obtained on intervention Days 1 and 5 to assess both acute and cumulative cortical responses to stimulation. EEG will be recorded with a 20-channel TMS-compatible cap arranged in the 10–20 system, using CP6 as the reference electrode and FC6 as the ground. Data will be acquired using an actiCHamp Plus amplifier (Brain Products GmbH, Germany), sampled at 50 kHz for TMS-evoked potentials and 5 kHz for resting-state and during-stimulation EEG (rs-EEG and iTBS-EEG).

#### TMS-evoked potential

Single-pulse TMS will be administered pre- and post-intervention on the first and last sessions of the Days 1 and 5 to investigate how HF-iTBS modulates cortical excitation and inhibition in the targeted regions, including the left dlPFC and the left IPL. TEP measurements will be collected using active or sham stimulation protocols aligned with the respective intervention conditions to preserve participant blinding throughout the study. All stimulation parameters follow established international safety guidelines (34). For TEP recording, stimulation will be delivered using the same handheld Cooled B65/AP coil connected to a MagPro XP Orange Edition stimulator (MagVenture, Farum, Denmark). Single pulses will be administered approximately every 2 seconds with 20% temporal jitter to reduce anticipatory effects. Stimulation intensity is set to 100% of the adjusted individual aMT, consistent with the intervention stimulation intensity. To minimize auditory-evoked contamination of TEPs, continuous white noise will be delivered through insert earphones during TMS to suppress the clicking sound of the coil.

### Data management

As mentioned, all data entered will be kept in the electronic case report form (eCRF) REDCap™ (Vanderbilt University, TN, USA). Each participant will be identified using an internal study-specific code. Where applicable, double data entry or verification will be applied, particularly for manually transcribed paper data in REDCap. MRI and EEG files will be only stored locally in pseudonymized form on secure host (DRCMR) servers, with access restricted to authorized study personnel. Servers are regularly backed up by the IT-department of the Capital Region of Denmark, ensuring data integrity. Quality of MRI and EEG recordings will be ensured by trained scanner and EEG operators, who perform immediate qualitative assessments to identify and document the presence of avoidable artifacts.

The sponsor-investigator will maintain a complete list of all randomized participants and a separate log of screened but not randomized individuals, including reasons for exclusion.

### Confidentiality

Data protection and confidentiality will comply with all applicable national and institutional regulations. Personal information will be stored separately from study data and linked only via coded identifiers. Paper records, including signed informed consent forms, will be stored in locked cabinets in restricted-access areas. Scanned consent forms will be stored in participants’ medical records (SP) and are not linked to the coded study database. Participants will be informed in writing that their data will be handled, stored, and analyzed in compliance with applicable data protection laws, and access to identifying information will be restricted to authorized personnel only.

### Plans for collection, laboratory evaluation and storage of biological specimens for genetic or molecular analysis in this trial/future use

No biological samples will be stored for future use or analysis. Urine samples obtained for pregnancy testing (where applicable) and drug screening will be used solely for point-of-care evaluation and disposed of immediately thereafter, without storage or further processing.

## Statistical methods

### Statistical methods for primary and secondary outcomes

Descriptive analyses of the data collected at each assessment point (baseline, post-treatment, and follow-up) will be conducted for all randomized participants. The primary outcome is the between-group difference in change in HAMD-6 score from baseline (T0) to treatment termination (F1), analyzed under the intention-to-treat (ITT) principle (62). The corresponding IPL-versus-sham contrast will constitute a secondary efficacy analysis. Persistence of treatment effects at four-week follow-up (T2) will be evaluated as a secondary outcome.

The effect of the intervention will be assessed using a linear mixed-effects model (LMM) (63) including time, treatment, and their interaction (time × treatment) as fixed effects (64). Baseline (T0) will be included as part of the repeated outcome measurements, with the primary treatment effect estimated from the treatment-by-time contrast from T0 to F1 for the active dlPFC versus sham groups. To account for repeated measures within individuals, a random intercept for each participant will be included. An unstructured covariance structure will be used for the repeated measurements; if this model does not converge, a simpler covariance structure will be selected based on model fit and convergence. Models will be estimated using maximum likelihood, allowing inclusion of participants with incomplete follow-up data under the missing-at-random assumption. The extent and patterns of missing outcome data will be summarized by treatment group. Sensitivity analyses of the primary outcome will assess the robustness of the findings to the handling of missing data and, where the extent of missingness permits, to plausible departures from the missing-at-random assumption.

Secondary outcomes including additional clinical scales, side-effect measures, and cognitive/motor assessments will be analyzed using similar mixed-effects models with fixed effects for time, treatment, and their interaction, and participant-level random intercepts. Categorical variables (e.g., adverse event presence, treatment response/remission) will be analyzed using chi-square tests or logistic regression (65).

The dlPFC-versus-sham HAMD-6 comparison constitutes the single confirmatory primary efficacy analysis. Key secondary clinical outcomes will be prioritized for interpretation, while the remaining secondary outcomes will be considered supportive and the neuroimaging and neurophysiological outcomes exploratory. Analyses beyond the primary efficacy comparison will be interpreted accordingly, with effect estimates and confidence intervals reported to support interpretation.

Exploratory analyses of brain imaging data will be conducted separately. Functional MRI data will be pre-processed using fMRIPrep (V 25.2.3) (66), followed by connectivity analyses in Nilearn Python package (67) and Statistical Parametric Mapping (SPM) software (68). A seed-to-voxel approach will be used with the sgACC as the seed region, and OFC, dlPFC, and IPL as regions of interest. Correction for multiple comparisons will be performed at the cluster or voxel level (p<0.05). Exploratory effective connectivity of the task-based functional MRI data will be assessed using dynamic causal modeling (DCM) in SPM, and associations between connectivity changes and clinical outcomes will be examined.

EEG data will be analyzed to evaluate changes in neural activity across groups (active vs. sham) and stimulation sites (dlPFC vs. IPL). Mixed-effects models will be applied to resting-state EEG measures, EEG responses during stimulation, and transcranial evoked potentials (TEPs), examining effects of time, treatment conditions, and stimulation site. Both spectral features (including periodic and non-periodic parameters) (69, 70) and TEP component characteristics (e.g., amplitude and latency) (71) will be compared across conditions. Associations between EEG features and clinical outcomes will be explored using correlational analyses.

### Interim analyses

No formal interim analyses of treatment effects are planned. Data processing and preliminary analyses may be conducted during the trial while treatment allocation remains blinded. All confirmatory analyses evaluating treatment efficacy will be performed only after completion of data collection and database lock. This approach preserves statistical integrity, minimizes bias, and prevents premature interpretation of treatment effects.

### Methods for additional analyses (e.g. subgroup analyses)

If relevant, exploratory *post hoc* analyses may be conducted to inform future hypotheses, but these will be clearly reported as such and interpreted with caution.

## Oversight and monitoring

### Composition of the coordinating center and trial steering committee

Each clinical site is managed by local investigators and research staff who oversee recruitment, intervention delivery and participant assessments, meeting regularly to monitor enrollment targets and protocol adherence. The coordinating center, based at DRCMR, will be responsible for central trial management, including coordination of intervention delivery, multimodal neuroimaging procedures (MRI, EEG, EMG), procedural harmonization across sites, and overall protocol compliance. Day-to-day trial operations will be managed jointly by the coordinating team and local site staff through routine communication and internal meetings.

### Composition of the data monitoring committee, its role and reporting structure

No independent Data Monitoring Committee will be established for this study, as the intervention and procedures are considered to pose minimal risk to participants. Although no formal external audits are planned, routine internal monitoring and auditing of trial procedures will be performed by local site staff and the coordinating center. These activities will ensure adherence to Good Clinical Practice principles (72), robust data governance, and ongoing protection of participant safety throughout the trial.

### Adverse event reporting and harms

Minor stimulation-related side effects may occur, such as scalp sensations or muscle twitches caused by peripheral co-activation during rTMS. These will be systematically assessed with the TMS-Sens-Questionnaire (34, 49) and documented in eCRF as outlined in this protocol.

Suicidality is prospectively monitored throughout the intervention week using HAMD-17 item 3, assessed daily after stimulation. Any clinically significant emergence or worsening of suicidal ideation is evaluated by the study physician and managed according to established clinical procedures. In the unlikely event of a suspected TMS-induced seizure, stimulation will be stopped immediately, and the participant will be managed according to established institutional TMS safety and emergency procedures.

A serious adverse event (SAE) is defined as any event that results in death, is life-threatening, requires hospitalization or prolongation of hospitalization, causes persistent or significant disability, or is otherwise deemed medically significant (72). All SAEs and any unexpected adverse reactions will be reported to the sponsor and relevant regulatory authorities, in accordance with regulatory timelines and local guidelines (73).

The study adheres to current international safety guidelines for MRI (74) and rTMS (34), and all AEs and SAEs will be followed until resolution, stabilization, or return to baseline.

### Plans for communicating important protocol amendments to relevant parties (e.g. trial participants, ethical committees)

An initial protocol amendment was implemented prior to participant enrollment to optimize assessment of timing, participant burden, and blinding procedures, as well as to enhance the clinical and physiological data collected.

The second protocol amendment was approved on 27 November 2025 (new case number:16-0302-29). This amendment introduced additional recruitment channels via TrialTree.dk and permitted distribution of project information within outpatient psychiatric clinics to enhance participant outreach. Furthermore, a structured referral pathway was added, ensuring that participants who do not achieve remission following the CoMPACT intervention are offered standard TMS therapy, which is routinely used in clinical psychiatric settings. These changes did not impact the intervention or the primary and secondary outcomes.

Any future protocol modifications will be promptly reported to Danish Medical Research Ethics Committee (VMK), ClinicalTrials.gov and communicated to all relevant collaborators and trial participants.

### Dissemination plans

Trial findings, regardless of outcome, will be disseminated through peer-reviewed publications and uploaded to public databases, including ClinicalTrials.gov and https://www.clinicaltrialsregister.eu. Results may also be shared via preprint platforms such as bioRxiv and presented at relevant scientific conferences. Dissemination strategies will be discussed with stakeholder representatives following data analysis. Co-authorship will comply with the ICMJE recommendations (Vancouver guidelines). If the clinical outcomes are favorable and implementation is feasible, training for mental health professionals in the CoMPACT protocol will be offered. Participants will be also informed about the main study findings through a plain-language summary distributed after completion of the trial.

## Discussion

Accelerated iTBS has demonstrated encouraging effects in reducing depressive symptoms in individuals with TRD (16). However, evidence is still limited, the underlying mechanisms are poorly understood, and the feasibility of delivering high-dose protocols over several hours per day poses practical challenges in clinical settings. This study aims to address these gaps by testing a more refined and feasible protocol that combines fewer sessions of HF-bursts of iTBS, personalized dosing, and testing a novel brain region in addition to the conventional target.

As CoMPACT integrates several modifications to previously described accelerated iTBS approaches, the present study evaluates the combined protocol as an integrated intervention and is not designed to assess the effects of individual protocol parameters separately. The goal is to evaluate whether this approach can deliver similar or improved outcomes compared with conventional protocols while being more practical and scalable for clinical implementation. In addition, the study integrates multimodal neuroimaging and neurophysiological assessments to investigate the neural mechanisms associated with treatment response. The trial will also explore associations between longitudinal changes in brain network function and clinical outcomes. Subsequently, this work is intended to support the development of more personalized, circuit-based brain stimulation therapies for treatment-resistant depression.

## Trial status

Participant recruitment is ongoing. The first enrollment took place in January 2025 and is expected to conclude in August 2028.

## Glossary

BCT: Brain-circuit-therapy
CoMPACT: Copenhagen Magnetic Personalized Accelerated Brain Circuit Therapy
DRCMR: Danish Research Centre for Magnetic Resonance
dlPFC: dorsolateral prefrontal cortex
EEG: Electroencephalography
ECG: Electrocardiography
ECT: Electro-convulsive therapy
FDA: Food and Drug Administration
HAMD-6: Hamilton Depression Scale (6 Items)
HAMD-17: Hamilton Depression Scale (17 Items)
IPL: Inferior parietal lobule
ITT: Intention to treat
LTP: Long term potentiation
MDD: Major depressive disorder
MDI: Major depression inventory
MINI: Mini Neuropsychiatric interview
MRI: Magnetic resonance imaging
fMRI: Functional magnetic resonance imaging
OFC: Orbitofrontal cortex
sgACC: Subgenual anterior cingulate cortex
SAINT: Stanford Accelerated Intelligent Neuromodulation Therapy
TBS: Theta-burst stimulation
TMS: Transcranial magnetic stimulation
TRD: Treatment-resistant depression
WHO-5: World Health Organization-Five Well-Being Index
SHAPS: Snaith-Hamilton Pleasure Scale
OASIS: Overall Anxiety Severity and Impairment Scale
VAMS: Visual Analogue Mood Scale
FAST: Functioning Assessment Short Test
PSQI: Pittsburgh Sleep Quality Index
TMSens_Q: TMS Sensation Questionnaire
SCIP: Screening for Cognitive Impairment in Psychiatry
COBRA: Cognitive Complaints in Bipolar Disorder Rating Assessment
ASAQ: Aarhus Side Effect Assessment Questionnaire
YMRS: Young Mania Rating Scale
SCWT: Stroop Color and Word Test
TMT: Trail Making Test A & B
9HPT: Nine-Hole Peg Test
TEX-Q: Treatment Expectation Questionnaire.
